# Anomalous insertion of anterior and posterior horns of medial meniscus. Case report

**DOI:** 10.1186/s12891-021-04696-6

**Published:** 2021-09-24

**Authors:** Pier Paolo Mariani, Michael J Battaglia, Guglielmo Torre

**Affiliations:** 1 Villa Stuart Sport Clinic-FIFA Medical Centre of Excellence, University of Rome Foro Italico, Rome, Italy; 2 Villa Stuart Sport Clinic-FIFA Medical Centre of Excellence, Rome, Italy; 3 Orthopaedic and Trauma Department, Campus Bio-Medico University of Rome, Rome, Italy

**Keywords:** Meniscus, Posterior Root, Anatomical Variant, Arthroscopy, Knee

## Abstract

**Background:**

Anatomical variations of the attachment of medial meniscus are a common finding. However, anomalies of the posterior horn are extremely rare. Only two cases of posterior root anomaly have been described prior to the routine use of arthroscopy for evaluation and treatment of meniscal pathology. In this report, we present an anomaly of both the anterior and posterior roots of the medial meniscus that posed both a diagnostic and therapeutic dilemma.

**Case presentation:**

The patient is young male soccer player who is currently 16 years of age and began having the atraumatic onset of pain and symptoms that limited performance starting at age 14 and was referred for failure of response to nonoperative treatment. Diagnostic arthroscopy revealed the presence of an anteromedial meniscofemoral ligament whereas the posterior root showed no bony attachment. The radiographic and arthroscopic findings are described. The clinical decision was made after to proceed with observation, reassurance, and gradual return to full activity with physiotherapy guidance.

**Discussion and conclusion:**

The absence of injury, the mild complaints reported by the patient, his age, skeletal immaturity, and remaining growth led us to adopt a conservative approach to treating this anatomic variant and currently the patient is able to participate fully in sports without symptoms or restrictions.

## Background

Anomalies of the medial meniscus are rare compared with those of the lateral meniscus. They include discoid variants, hypoplasia of anterior horn, and anomalous attachment of the anterior horn. Usually, the most common anomaly of medial meniscus is related to the anterior insertion that often shows anatomic variability [1–3]. However, anomalous attachment of posterior horn has been reported in only two cases [4, 5]. In the first case [4], the authors described the presence of a fibro-cartilaginous fold covering the medial femoral condyle. The posterior horn was described as normal. In the second case [5], the authors described the presence of a fibrous band arising from the posterior horn and inserting to femoral insertion of the ACL. Both cases were described prior to the common use of arthroscopy. Recently, Sadigursky et al. [6] reported a case of abnormality in medial and lateral posterior horns. We present the first report of an anomaly of both the anterior and posterior roots of the medial meniscus, documented by MRI and confirmed by arthroscopy.

## Case presentation

**Fig. 1:**
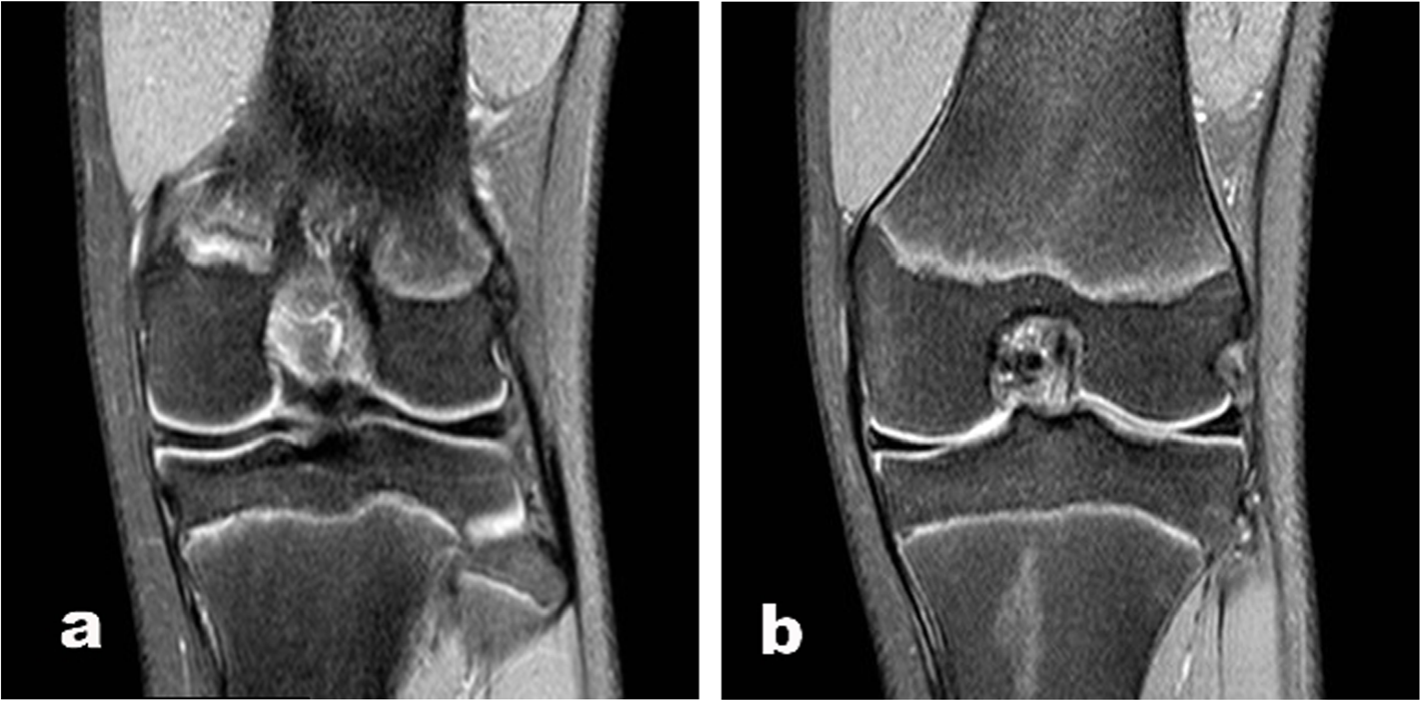
Coronal T2-weighted MRI scan shows absence of the posterior medial root (**A**) and on the coronal image no clear signs of extrusion were detected (**B**)

**Fig. 2:**
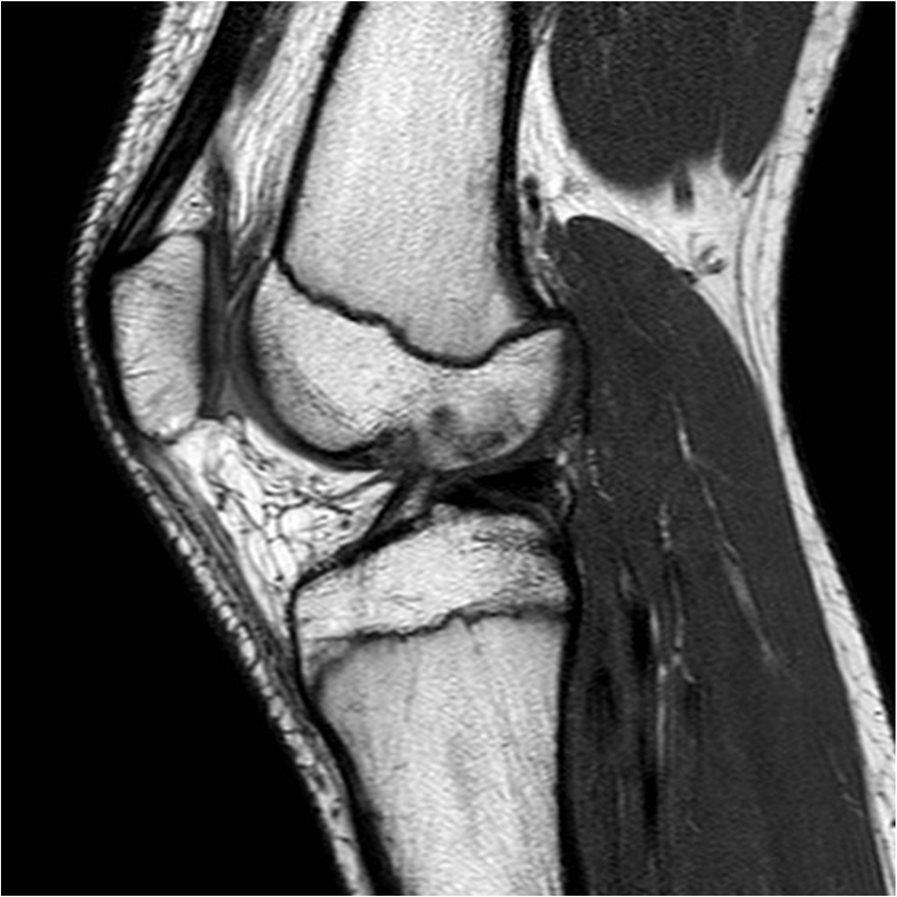
The MRI shows abnormal band originated from the anterior horn of medial meniscus directed to the femoral insertion of the ACL (red arrow)

**Fig. 3:**
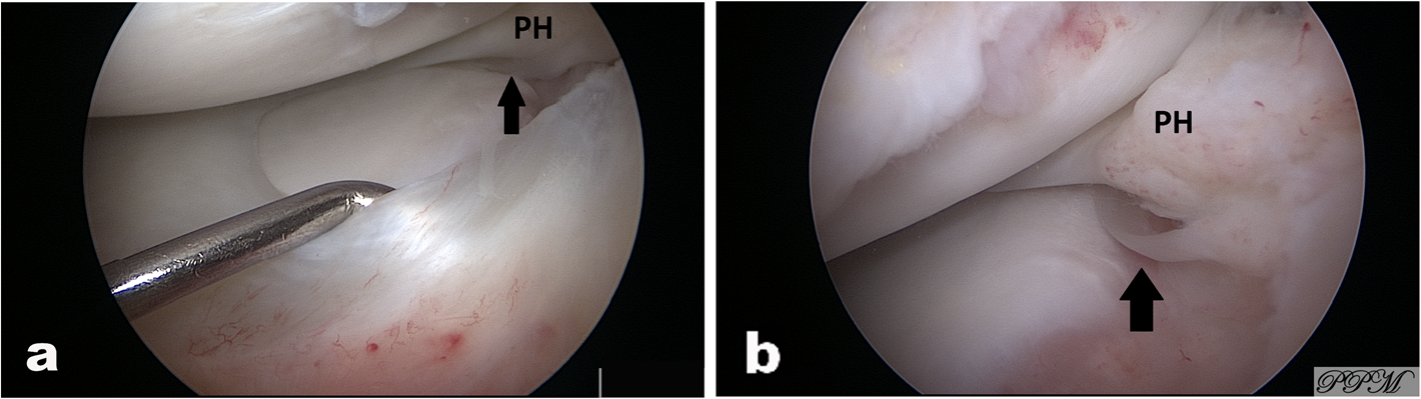
Arthroscopic finding of the left knee joint. In (**A**) view of anterior horn and the probe indicates the presence of anterior meniscofemoral ligament whereas on the back it is visible the posterior horn (ph) present but raised up for absence of bony attachment. In (**B**) detail of posterior root with presence of some fibrous tissue connecting the posterior horn to posterior cruciate ligament and a thin band below the undersurface of the meniscus (arrow)

A 14-year old male soccer player was referred to our orthopedic tertiary care referral facility as a result of failure to respond to non-operative management. He first experienced pain on the medial side two years prior that was noted after sports activities although no clear injury was identified. The knee was intermittently painful and with time these symptoms progressively worsened despite several attempts at non-operative management to include anti-inflammatories and physical therapy. The symptoms limited the ability of the patient to participate in sports. Physical examination revealed no effusion, complete range of motion, and no signs of meniscal pathology. There was no tenderness to palpation of the medial or lateral joint line, a negative McMurray, and a normal ligamentous examination. There was no pain with hyperextension, and the only positive examination finding was mild pain on hyperflexion. Radiographs were normal. Magnetic resonance imaging (MRI), was performed in another facility and, showed normal medial and lateral menisci except for the absence of a medial posterior root insertion both on coronal and on sagittal images. There was no evidence of meniscal extrusion or a meniscal ghost sign (Fig. 1). On sagittal magnetic resonance images, the presence of thin low-signal band was identified just anterior to the ACL (Fig. 2). Diagnostic arthroscopy was performed secondary to failure of nonoperative management and revealed a white rounded band that was noted to extend from the anterior horn of the medial meniscus to the intercondylar notch adjacent to the femoral insertion of the ACL (Fig. 3A). This band was clearly a distinct structure from the ACL with probing. Next careful examination of medial compartment revealed that the meniscus did not have evidence of tearing or degenerative changes and appeared to be floating into the medial compartment without the typical anterior bony attachment. The posterior horn showed absence of any firm bony attachment as well. Some fibers were detected below the anterior aspect of posterior cruciate running towards the lateral compartment (Fig. 3B). No chondral pathology was detected. Because of absence of meniscal lesions or other intrarticular abnormalities, we elected close observation rather than surgical intervention. The patient made rapid recovery after diagnostic arthroscopy and returned to his soccer activity after 2 months without any pain, discomfort, or limitation of activity. At 4-year follow-up, he had no symptoms or abnormal findings and he was still playing soccer at a youth competitive level. On the follow-up MRI there no meniscal or chondral lesions and no significative change of the appearance of the meniscal roots from the index MRI.

## Discussion and conclusion

There were two different challenges that needed to be addressed in treating this patient. The first one was the diagnostic issue secondary to the vague and nonspecific symptoms of the patient and lack of clear identifiable objective pathology. The only positive finding was the presence of an anomalous anterior horn insertion on MRI scan. The anatomy of the anterior meniscal root has been described by Berlet and Fowler [1]: they distinguished four different types, three with bony insertion and the fourth type inserting into the soft type (intermeniscal ligament). In addition to differences of anatomical insertions of anterior medial meniscus horn, in 1993 McCormack and McGrath [7] described a further type of the anterior horn attachment without bony insertion but with a strong ligament-like tissue attachment to the posterolateral wall of the femoral intercondylar fossa, covering the anterior portion of the ACL. They named this anomaly as the anteromedial meniscofemoral ligament (AMMFL). Following this first report, many other observations of this anatomical variant have been described [8–12]. The true incidence of AMMFL is not yet known. Rainio et al. [13] reported an incidence of 1.2 % in 987 consecutive arthroscopies. Cha et al. [14] in a cohort of 1,326 arthroscopic examinations reported the incidence to be 2.3 %. More recently, Kim and Joo [15] have reported that in their patient population, the presence of AMMFL was detected only in 0.5 % of cases. The ability of the MR scan to detect an anomalous insertion of the medial meniscus is challenging [16] with a 74 % false-positive rate for anterior horn tears [17]. Moreover, the AMMFL may easily be confused as fibers of the ACL [18]. The senior author has performed over 4000 arthroscopies in the past 20 years and only identified 0.3 % which is consistent with published literature. In all cases described in the literature, the AMMFL was an incidental finding and its clinical significance remains unknown. The symptoms are often vague and often referred to the anterior knee. The cause of symptoms is hypothesized to be related to impingement against the femoral condyle [19] or to abnormal mobility of the anterior horn that may predispose the meniscus to a possible rupture or to progressive degeneration [7]. The pain is often reported after athletic activity, but a clear traumatic incident is seldom reported.

In our case, the diagnostic dilemma was complicated by the concomitant lack of attachment of the posterior horn of the meniscus to the tibia. On index MRI, because of presence of an intact posterior horn, the classic signs of meniscal root pathology, such as meniscal extrusion or meniscus ghost, were absent. When probed, it was possible to detect only few fibers running in front of the anterior portion of posterior cruciate ligament toward the posterior horn of the lateral meniscus. These fibers could be interpreted as a rare posterior transverse ligament or meniscomeniscal ligament [20]. Apart from these fibers, no other bands or anomalies of the posterior horn were detected. In the literature, anomalous attachment of the posterior horn is extremely rare and only two cases have been reported. In 1963 Riach and Phares [4] described a case in which the posterior horn continued through a fold to cover the articular surface of the medial femoral condyle. In 1998, Bhagava and Ferrari [5] described a case of an anomalous band from the posterior horn area of the medial meniscus to insert into the midportion of anterior cruciate ligament.

For this particular patient, the main problem we faced was the choice of treatment given the intermittent symptoms. Generally, an AMMFL does not seem to be related to knee symptoms, and because is often an incidental finding it may be left in situ. However, in some reports, the ligament has been cut and removed with reported improvement of symptoms [13, 19, 21, 22]. It is our opinion that is difficult to support resection as an option in a young patient with remaining growth given the lack of consensus in the literature. We agree with other authors who consider the AMMFL to have a biomechanical effect as a ligamentous anchor of the anterior horn [10, 15]. In our particular case, where a combined anomaly of both horns was present, the AMMFL was the only meniscal attachment. Therefore, to remove this isolated fibrous insertion could potentially increase hypermobility of the medial meniscus and therefore be counterproductive.

Because of the young age of our patient with open physes there were potential deleterious effects that could result from the absence of definitive attachment of anterior or posterior horn. The absence of injury, the mild complaints reported by the patient, his age, skeletal immaturity, and remaining growth led us to adopt a conservative approach to treating this anatomic variant. At the present time, the patient still actively practices and competes in soccer with no symptoms that limit participation.

## Data Availability

No dataset analysis was carried out for the current study. Clinical and radiological reports are available from the corresponding author on reasonable request.

## Abbreviations

ACL: anterior cruciate ligament.
MRI: magnetic resonance images.
AMMFL: anteromedial meniscofemoral ligament.
